# Preovulatory follicular fluid secretome added to in vitro maturation medium influences the metabolism of equine cumulus-oocyte complexes

**DOI:** 10.1186/s12917-024-04129-1

**Published:** 2024-06-25

**Authors:** Marcos Luis-Calero, José Manuel Ortiz-Rodríguez, Pablo Fernández-Hernández, Carmen Cristina Muñoz-García, Eva Pericuesta, Alfonso Gutiérrez-Adán, Federica Marinaro, Nieves Embade, Ricardo Conde, Maider Bizkarguenaga, Óscar Millet, Lauro González-Fernández, Beatriz Macías-García

**Affiliations:** 1 Departamento de Medicina Animal, Grupo de Investigación Medicina Interna Veterinaria (MINVET), Facultad de Veterinaria, Instituto de Investigación INBIO G+C, Universidad de Extremadura, Av. de la Universidad s/n, Cáceres, 10004 Spain; 2 Department of Veterinary Medical Sciences (DIMEVET), University of Bologna, Via Tolara di Sopra 50, Bologna, 40064 Ozzano dell’Emilia Italy; 3 Departamento de Reproducción Animal, INIA-CSIC, Av. Puerta de Hierro 18, Madrid, 28040 Spain; 4 Precision Medicine and Metabolism Laboratory, CIC bioGUNE, Basque Research and Technology Alliance (BRTA), Parque Tecnológico de Bizkaia, 801 A Building, Derio, 48160 Bizkaia Spain; 5 Departamento de Bioquímica y Biología Molecular y Genética, Grupo de Investigación Señalización Intracelular y Tecnología de la Reproducción (SINTREP), Instituto de Investigación INBIO G+C, Facultad de Veterinaria, Universidad de Extremadura, Av. de la Universidad s/n, Cáceres, 10004 Spain

**Keywords:** COCs, Secretome, Oocyte, *In vitro* maturation, Horse

## Abstract

**Background:**

In vitro embryo production is a highly demanded reproductive technology in horses, which requires the recovery (in vivo or *post-mortem*) and in vitro maturation (IVM) of oocytes. Oocytes subjected to IVM exhibit poor developmental competence compared to their in vivo counterparts, being this related to a suboptimal composition of commercial maturation media. The objective of this work was to study the effect of different concentrations of secretome obtained from equine preovulatory follicular fluid (FF) on cumulus-oocyte complexes (COCs) during IVM. COCs retrieved in vivo by ovum pick up (OPU) or *post-mortem* from a slaughterhouse (SLA) were subjected to IVM in the presence or absence of secretome (Control: 0 µg/ml, S20: 20 µg/ml or S40: 40 µg/ml). After IVM, the metabolome of the medium used for oocyte maturation prior (Pre-IVM) and after IVM (Post-IVM), COCs mRNA expression, and oocyte meiotic competence were analysed.

**Results:**

IVM leads to lactic acid production and an acetic acid consumption in COCs obtained from OPU and SLA. However, glucose consumption after IVM was higher in COCs from OPU when S40 was added (Control Pre-IVM vs. S40 Post-IVM: 117.24 ± 7.72 vs. 82.69 ± 4.24; Mean µM ± SEM; *p* < 0.05), while this was not observed in COCs from SLA. Likewise, secretome enhanced uptake of threonine (Control Pre-IVM vs. S20 Post-IVM vs. S40 Post-IVM: 4.93 ± 0.33 vs. 3.04 ± 0.25 vs. 2.84 ± 0.27; Mean µM ± SEM; *p* < 0.05) in COCs recovered by OPU. Regarding the relative mRNA expression of candidate genes related to metabolism, Lactate dehydrogenase A *(LDHA)* expression was significantly downregulated when secretome was added during IVM at 20–40 µg/ml in OPU-derived COCs (Control vs. S20 vs. S40: 1.77 ± 0.14 vs. 1 ± 0.25 vs. 1.23 ± 0.14; fold change ± SEM; *p* < 0.05), but not in SLA COCs.

**Conclusions:**

The addition of secretome during in vitro maturation (IVM) affects the gene expression of LDHA, glucose metabolism, and amino acid turnover in equine cumulus-oocyte complexes (COCs), with diverging outcomes observed between COCs retrieved using ovum pick up (OPU) and slaughterhouse-derived COCs (SLA).

**Supplementary Information:**

The online version contains supplementary material available at 10.1186/s12917-024-04129-1.

•Part of the data included were presented at The International Symposium on Equine Reproduction (ISER) held in 2023 in Foz de Iguaçu (Brasil): “Ortiz-Rodriguez, J. M., Fernández-Hernández, P., Luis-Calero, M., Spinaci, M., Bucci, D., Macías-García, B., & González-Fernández, L. (2023). Influence of secretome obtained from preovulatory follicular fluid on energy metabolism and meiotic competence of equine cumulus–oocyte complexes. Journal of Equine Veterinary Science, 125, 104670”.

## Background

Assisted reproductive technologies (ARTs) are being increasingly requested by the equine industry to enhance the mare’s reproductive efficiency. Among ARTs, in vitro embryo production (IVEP) is of great interest as it optimizes the number of offspring that a mare can produce during her lifetime [1]. In mammals, IVEP can be performed by conventional in vitro fertilization (IVF) or by intracytoplasmic sperm injection (ICSI) both of which require oocyte retrieval (in vivo or *post-mortem*) [2]. It is worth mentioning that even when both oocyte sources are used indistinctly, differences exist in their meiotic competence, degeneration during IVM and early embryo reabsorption rates [3]. Once harvested, oocytes are subjected to in vitro maturation (IVM) in commercial media added with hormones and other additives, but its composition largely differs from the follicular fluid (FF) [4]. Oocyte maturation is influenced by FF [5] and depends on the bi-directional communication between the oocyte and the surrounding cumulus cells through gap junctions, forming the so-known cumulus-oocyte complex (COC) [6–8]. This phenomenon involves a series of finely synchronized nuclear and cytoplasmic changes, such as chromatin remodelling, cytoplasmic lipid droplet aggregation, and mitochondrial redistribution among others [9].

Granulosa cells (mural and cumulus cells) are responsible for FF production [10]. FF is composed by proteins, lipids, extracellular vesicles (EVs), metabolites, hormones, enzymes, DNA, and mRNA and its composition vividly varies depending on the phase of the estrous cycle [11]. The biologically active fraction of FF is known as “secretome” [12] and plays a core role during oocyte maturation, determining the oocyte’s meiotic and developmental competence [13]. The main difference between a biological fluid and its secretome is that the latter refers specifically to the subset of proteins secreted or released by cells into the extracellular space. Importantly, the composition of the secretome can vary significantly depending on the secretome´s source [14]. As previously mentioned, equine oocytes are generally retrieved in an immature stage and must undergo IVM. However, this step is inefficient, as even when maturation rates exceed 60%, the final blastocyst yield is still low, varying between 11% and 29% in clinical settings [1, 15, 16]. This limited efficiency could be in part attributed to the vivid differences existing in the composition of equine FF and the currently used IVM media, which negatively impact the oocytes’ meiotic and developmental competence [4]. It has been demonstrated that current IVM media used for equine oocytes fail to meet the metabolic needs of the female gamete, which may explain in part their low developmental competence and high reabsorption rates during early pregnancy [17]. A well-balanced oocyte energy metabolism profoundly influences various aspects of cytoplasmic and nuclear maturation, along with the subsequent developmental competence of embryos [18]. Therefore, the maintenance of homeostasis between glucose and fatty acid metabolism in oocytes is known to determine the oocyte’s fate [18] and some research has already been conducted in horses regarding glucose metabolism [17] and oocyte´s lipid content [19]. On the other hand, different studies have aimed to increase IVM success using diverse additives in the IVM medium including native follicular fluid, hormones, or metabolites among others, but these attempts have not resulted in significant improvements in maturation rates [11, 20–22].

Hence, we aimed to use secretome obtained from equine preovulatory FF as an additive during IVM. After maturation in presence or absence of FF secretome, we evaluated the metabolic activity of COCs through metabolomic analysis of IVM supernatants. Additionally, we assessed the oocyte meiotic competence of oocytes and examined the expression of candidate genes involved in glucose and lipid metabolism in equine COCs. This study was conducted separately using oocytes retrieved in vivo or *post-mortem*, as our hypothesis was that oocyte source (OPU or *post-mortem*) may influence the COCs’ metabolic requirements.

## Results

### Equine oocyte maturation and degeneration rates

The use of secretome either at 20–40 µg/ml during IVM did not influence the proportion of oocytes exhibiting germinal vesicle (GV), metaphase I (MI), metaphase II (MII) or degenerated (DEG) chromatin compared against control, regardless of the oocyte source (OPU or SLA; Supplementary Tables 1 and 2) coinciding with our previous report [12, 23].

### Effect of secretome supplementation on COCs’ metabolism

Metabolite identification was conducted in the maturation medium before and after IVM to assess the influence of secretome supplementation on COC’s metabolism depending on their origin (OPU or SLA). Nuclear magnetic resonance (NMR) analysis detected lactic acid, glucose, tyrosine, glycerol, succinic acid, pyruvic acid, glutamic acid, acetic acid, alanine, 3-hydroxyisovaleric, valine, isoleucine, leucine, and threonine in all samples from the SLA and OPU groups (Tables 1 and 2). Statistical analysis of the media prior to IVM for control, S20 and S40 groups resulted in no significant differences among them (data not shown), indicating that secretome addition at the dosages tested does not alter the concentration of the metabolites identified.

#### *Post-mortem* retrieved oocytes

The supernatant retrieved after IVM in all treatments (control, S20 and S40) showed a significant increase in lactic acid concentration and a significant decrease in acetic acid concentration compared to the control medium prior to IVM (Table 1). No significant differences were observed for the rest of the metabolites (Table 1; *p* > 0.05).

**Table 1:** Metabolite concentration (µM) in maturation medium prior (Pre-IVM) or after (Post-IVM) of equine COCs obtained from SLA subjected to IVM in the presence or absence of secretome

SLA	CTR Pre-IVM	CTR Post-IVM	S20 Post-IVM	S40 Post-IVM
**Lactic acid**	110.46 ± 4.87	275.44 ± 40.5 **	275.88 ± 23.33 **	273.85 ± 43.81 **
**Glucose**	106 ± 6.04	68 ± 8.45	68.43 ± 7.71	69.37 ± 9.96
**Tyrosine**	22.04 ± 0.58	20.49 ± 1.23	24.91 ± 0.62	21.41 ± 2.25
**Glycerol**	74.2 ± 6.67	76.5 ± 5.81	78 ± 7.62	77.05 ± 7.33
**Succinic acid**	5.78 ± 0.17	5.39 ± 0.25	5.42 ± 0.39	5.37 ± 0.08
**Pyruvic acid**	0.93 ± 0.21	3.1 ± 1.28	2.71 ± 0.78	3.01 ± 0.88
**Glutamic acid**	25.54 ± 1.57	31.92 ± 2.11	31.72 ± 1.02	30.34 ± 1.83
**Acetic acid**	17.52 ± 0.85	7.52 ± 1.69*	8.58 ± 1.52*	8.89 ± 1.65*
**Alanine**	18.62 ± 0.99	22.05 ± 2.44	22.27 ± 0.96	23.79 ± 0.14
**3-hydroxyisovaleric**	1 ± 0.17	0,92 ± 0,19	1 ± 0.17	1.1 ± 0,13
**Valine**	9.5 ± 0.68	10.95 ± 1.03	10.91 ± 0.67	10.09 ± 1.21
**Isoleucine**	12.85 ± 1.22	12.27 ± 1.33	11.85 ± 0.48	10.82 ± 0.74
**Leucine**	38.79 ± 1.5	33.49 ± 0.76	35.84 ± 2.2	35.52 ± 0.97
**Threonine**	4.03 ± 0.09	2.52 ± 0.19	2.4 ± 0.23	2.79 ± 0.34

Equine COCs were subjected to IVM in the absence (CTR) or presence of secretome from follicular fluid at 20 µg/ml (S20) or 40 µg/ml (S40). Treatments marked within the same row means that significant differences exist (*p < 0.05; **p < 0.01) compared to CTR Pre-IVM

#### OPU retrieved oocytes

Addition of FF secretome during IVM resulted in a significant increase in lactic acid concentration post-IVM for the S20 and S40 groups compared to the control medium prior IVM (Table 2; *p* < 0.05). Furthermore, all treatments displayed a significant decrease in acetic acid concentration post-IVM compared to the control medium prior IVM (Table 2; *p* < 0.05). Glucose concentration significantly decreased in COCs supplemented with secretome at 40 µg/ml compared to the control medium prior IVM (Control Pre-IVM vs. S40 Post-IVM: 117.24 ± 7.72 vs. 82.69 ± 4.24 µM; Mean ± SEM) (Table 2; *p* < 0.05). Likewise, threonine and acetic acid concentration significantly varied after IVM in the secretome-supplemented groups compared to the control medium prior IVM (Threonine: Control Pre-IVM vs. S20 Post-IVM vs. S40 Post-IVM: 4.93 ± 0.33 vs. 3.04 ± 0.25 vs. 2.84 ± 0.27 µM; Acetic Acid: Control Pre-IVM vs. S20 Post-IVM vs. S40 Post-IVM: 19.07 ± 1.16 vs. 15.71 ± 1.05 vs. 13.64 ± 0.38) (Table 2; *p* < 0.05). No statistically significant differences were observed for the rest of the metabolites (*p* > 0.05).

**Table 2:** Metabolite concentration (µM) in maturation medium prior (Pre-IVM) or after (Post-IVM) of equine COCs obtained by OPU subjected to IVM in the presence or absence of secretome

OPU	CTR Pre-IVM	CTR Post-IVM	S20 Post-IVM	S40 Post-IVM
**Lactic acid**	101.4 ± 6.47	161.68 ± 21.63	221 ± 24.87 ****	245.87 ± 11.84 ****
**Glucose**	117.24 ± 7.72	99.78 ± 3.34	103.41 ± 5.35	82.69 ± 4.24 ***
**Tyrosine**	23.81 ± 1.8	24.49 ± 1.56	26.58 ± 1.09	22.16 ± 1.07
**Glycerol**	74.99 ± 5.73	83.38 ± 5.33	68.75 ± 5.36	64.24 ± 3.48
**Succinic acid**	6.05 ± 0.39	6.61 ± 0.64	6.88 ± 0.21	6.56 ± 0.41
**Pyruvic acid**	1.03 ± 0.13	1.19 ± 0.08	1.13 ± 0.21	1.1 ± 0.17
**Glutamic acid**	30.48 ± 2.18	33.48 ± 3.15	33.72 ± 2.32	30.54 ± 2.01
**Acetic acid**	19.07 ± 1.16	15.41 ± 0.79 *	15.71 ± 1.05 *	13.64 ± 0.38 ***
**Alanine**	20 ± 1.3	22.26 ± 2.18	22.01 ± 0.79	20.39 ± 0.8
**3-hydroxyisovaleric**	1.04 ± 0.16	0.93 ± 0.09	0.81 ± 0.07	0.77 ± 0.12
**Valine**	12.02 ± 0.96	10.09 ± 0.67	11.38 ± 0.8	10.09 ± 0.64
**Isoleucine**	13.46 ± 1.21	13.51 ± 0.93	13.26 ± 0.46	12.52 ± 0.41
**Leucine**	41.73 ± 2.33	44.42 ± 2.74	41.23 ± 0.65	37.63 ± 0.99
**Threonine**	4.93 ± 0.33	3.88 ± 0.19	3.04 ± 0.25 *	2.84 ± 0.27 **

Equine COCs were subjected to IVM in the absence (CTR) or presence of secretome from follicular fluid at 20 µg/ml (S20) or 40 µg/ml (S40). Treatments marked within the same row means that significant differences exist (*p < 0.05; **p < 0.01; ***p < 0.001; ****p < 0.0001) compared to CTR Pre-IVM

### Effect of secretome supplementation on candidate gene mRNA expression

**Fig. 1:**
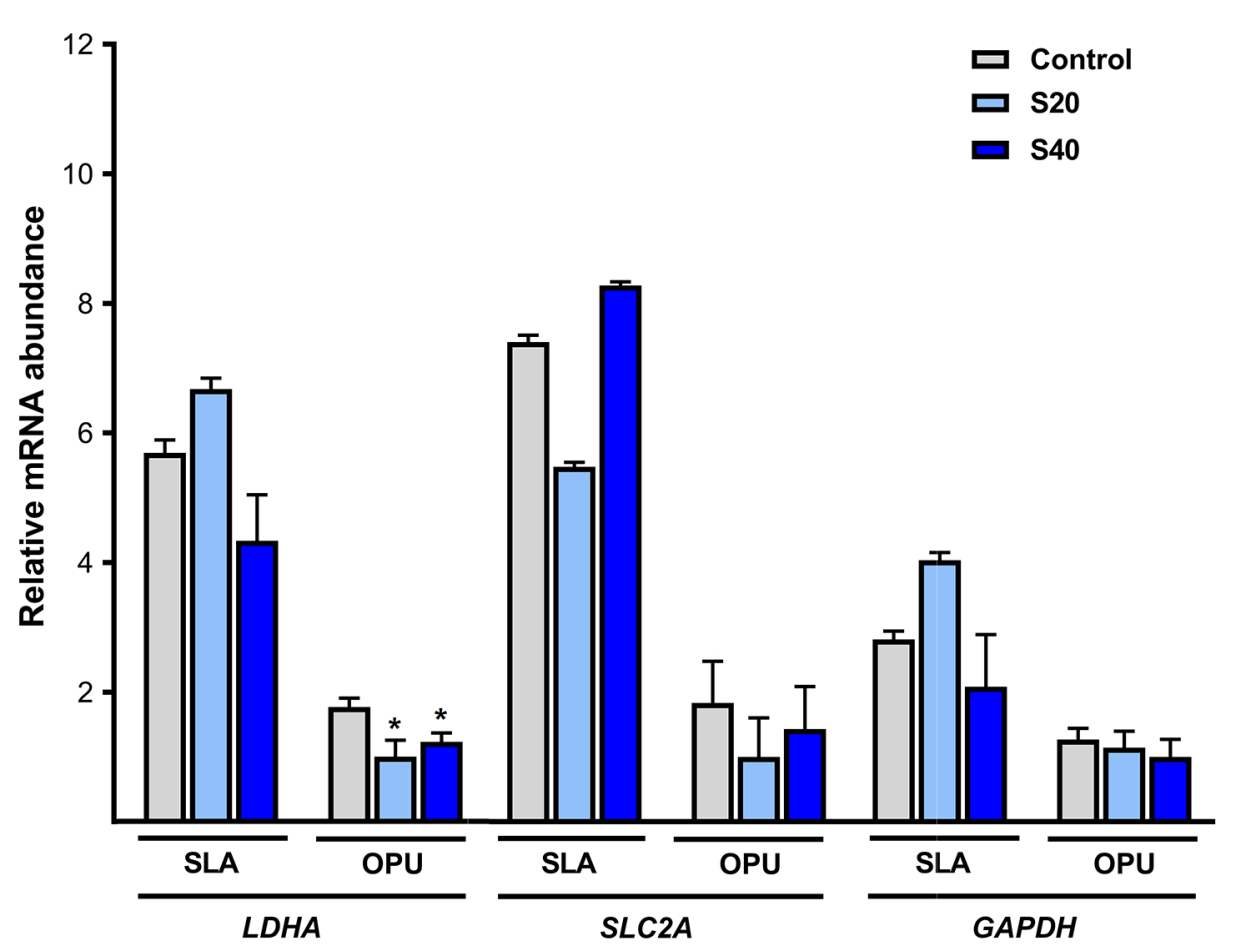
Relative mRNA abundance of genes related to glucose metabolism in equine COCs retrieved in vivo (OPU) or *post-mortem* (SLA) and matured in the absence (CTR) or the presence of secretome retrieved from preovulatory follicular fluid at 20 µg/ml (S20) or 40 µg/ml (S40). The expression of target genes was normalized against that of H2A histone family, member Z (*H2AFZ*), used as a housekeeping gene. Lactate dehydrogenase A (*LDHA*); Solute carrier family 2 member (*SLC2A*) and Glyceraldehyde-3-phosphate dehydrogenase (*GAPDH*). Data are expressed as the fold change ± SEM of three replicates. Bars representing a single gene marked with (*) differ significantly between treatments (CTR, S20 or S40) in the same group (SLA or OPU) (*p* < 0.05)

**Fig. 2:**
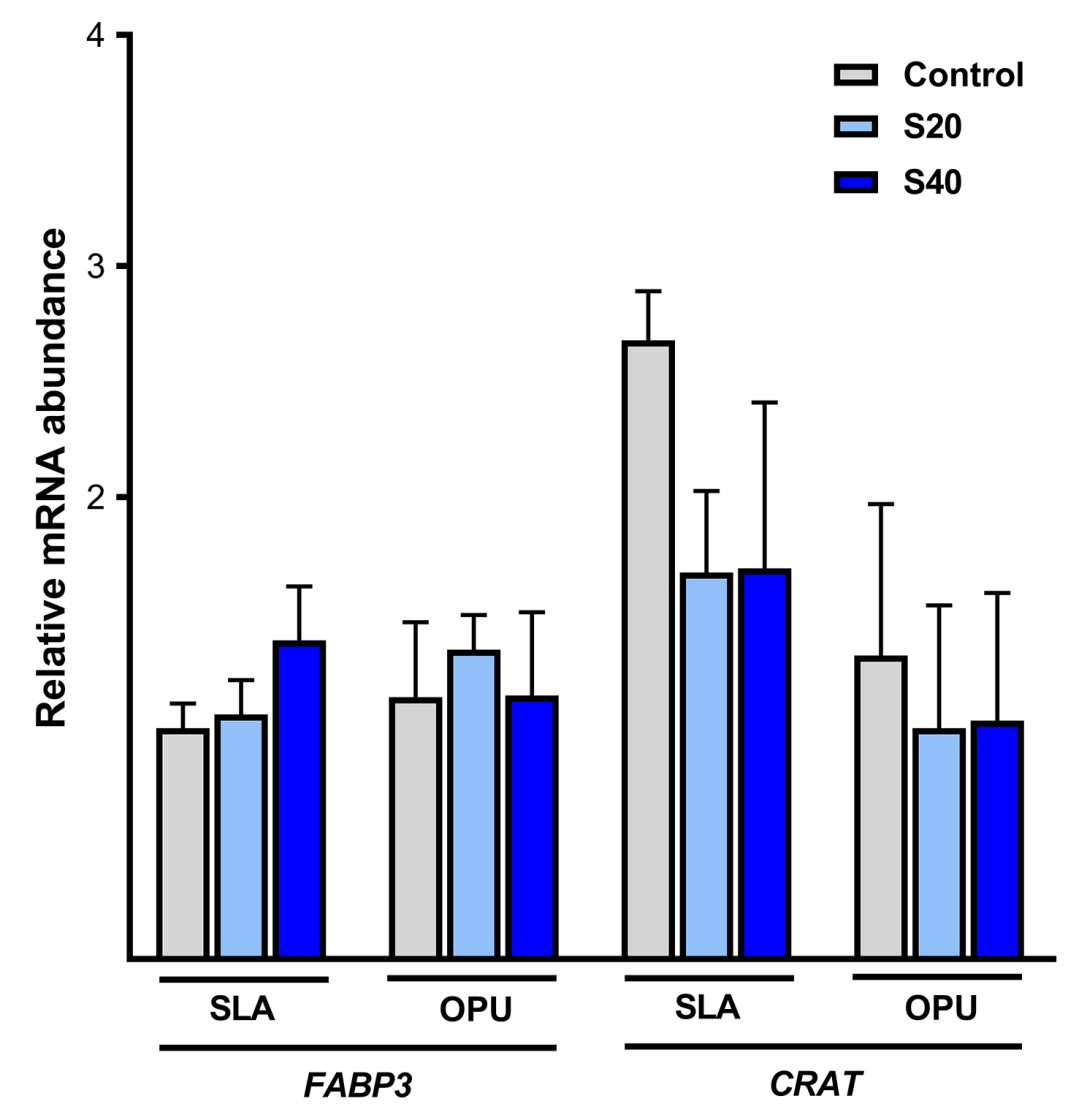
Relative mRNA abundance of genes related to lipid metabolism in equine COCs retrieved in vivo (OPU) or *post-mortem* (SLA) and matured in the absence (CTR) or the presence of secretome retrieved from preovulatory follicular fluid at 20 µg/ml (S20) or 40 µg/ml (S40). The expression of target genes was normalized against that of H2A histone family, member Z (*H2AFZ*), used as a housekeeping gene. Fatty acid binding protein 3 (*FABP3*) and Carnitine acetyltransferase (*CRAT*). Data are expressed as the fold change ± SEM of three replicates. No statistically significant differences were observed (*p* > 0.05)

In the last set of experiments, the relative mRNA abundance of genes related to glucose metabolism (Lactate dehydrogenase A, *LDHA*; Solute carrier family 2 member, *SLC2A*; and Glyceraldehyde-3-phosphate dehydrogenase, *GAPDH*) and lipid metabolism (Fatty Acid Binding Protein 3, *FABP3*; and Carnitine acetyltransferase, *CRAT*) was studied in equine COCs retrieved from OPU and SLA groups after IVM. In the SLA group, no significant differences were observed in the expression of any of the candidate genes studied, regardless of secretome addition (Figs. 1 and 2). Conversely, in the OPU group a significant decrease in the relative mRNA expression of *LDHA* was observed when secretome was added at 20–40 µg/ml compared to the control (Control vs. S20 vs. S40: 1.77 ± 0.14 vs. 1 ± 0.25 vs. 1.23 ± 0.14; fold change ± SEM) (*p* < 0.05; Fig. 1). However, no significant differences were observed for the rest of the genes studied (*p* > 0.05) (Figs. 1 and 2).

## Discussion

The success of IVM of equine oocytes is limited compared to other domestic species as there is no strict selection of oocytes prior maturation [24]. Recently, it has been described an IVM protocol in which EVs extracted from FF of small follicles enhanced nuclear maturation rates of equine COCs exhibiting compact cumulus [25]. However, EVs exhibit a large degree of heterogeneity in their size, biogenesis, and bioactive cargo, showing differences according to the follicular stage [26]. As previously mentioned, EVs are just a fraction of the so-known secretome in which other molecules such as soluble factors are present playing a pivotal role in oocyte maturation, determining the oocyte´s meiotic and developmental competence [13].

Our data revealed that secretome supplementation did not induce a significant increase in MII rate in oocytes from OPU or SLA, although a tendency for higher MII rate was observed associated with secretome addition after IVM. Furthermore, the percentage of degenerated oocytes was similar between treatments and their own control, showing that secretome from preovulatory FF used at the concentrations and conditions here described does not exert a deleterious effect on equine oocytes. In our experimental setting, two types of oocyte retrieval and holding protocols were used: OPU oocytes were immediately placed to IVM while SLA oocytes were held overnight prior IVM. Hence, higher degeneration rates and lower GV chromatin configuration were observed in COCs from SLA group coinciding with previous reports that attribute this increase to the storage of the ovaries for their transport from the abattoir [24]. A recent study on equine cloning, reported that when overnight holding was performed prior IVM from SLA- and OPU-derived oocytes, a higher proportion of oocytes from SLA group reached the MII stage compared to OPU group which does not match our findings. This difference was attributed to the fact that a higher number of COCs were recovered from the slaughterhouse and the elimination of approximately 15% of the retrieved COCs due to few cumulus cell clusters or the sole presence of the corona radiata [27]. This pre-selection was not done in our experimental setting, possibly explaining why OPU and SLA retrieved oocytes exhibit similar maturation rates in our work.

In relation to the metabolites identified, we observed a significant increase in lactic acid production after IVM in all treatments (CTR, S20 and S40) compared to the control prior IVM. This enhanced lactic acid production after IVM was paralleled by non-significant glucose depletion after IVM in SLA retrieved oocytes. Interestingly, in OPU-derived oocytes, a statistically significant reduction in glucose levels after IVM was observed only when IVM medium was supplemented with 40 µg/ml secretome. This drop could be related to an enhanced glucose uptake promoted by secretome as previously demonstrated in HepG2 cells in which addition of secretome retrieved from mesenchymal stem cells promoted the translocation of the glucose transporter 4 and thus, glucose internalization [28]. A recent study regarding energy metabolism in equine COCs during IVM established that approximately 95% of glucose consumption results in lactic acid production [17], which is coincident with our observations, as lactic acid concentration consistently doubles after IVM in the present work. Glucose can be transported into cells by SLC2A, a glucose membrane transporter whose expression has been previously described in equine COCs [29]. In our study, *SLC2A* expression was not influenced by secretome addition. Besides, core differences between competent and incompetent bovine oocytes have recently been described, establishing that *SLC2A* expression does not differ, but *GADPH* and *LDHA* are upregulated in incompetent oocytes [30]. In our work, COCs from OPU exhibited a lower expression of *LDHA* compared to the control in the S20 and S40 groups, which could be related to an enhanced developmental competence of the resulting COCs, as previously shown in bovine [30]. Also, the expression of *GAPDH* (an essential component of the glycolytic pathway) [31] showed no significant differences despite secretome addition.

On the other hand, when the supernatants of OPU-derived COCs were retrieved after IVM, a decrease in acetic acid concentration was observed. A recent study has positively correlated acetic acid metabolism in cumulus cells with oocyte maturation, as acetic acid is metabolized to acetyl-CoA enabling energy production by the Krebs cycle as a complement for acetyl-CoA production by the classical glycolytic pathway [32] coinciding with our results. Furthermore, equine oocytes present a large quantity of endogenous lipids that upon β-oxidation, result in ATP production. It is known that the oocyte´s and cumulus cells’ metabolism and ATP levels are associated with oocyte quality, developmental competence, and embryo health [33]. However, in our setting, no differences were observed among Fatty acid binding protein 3 (*FABP3*) and Carnitine acetyltransferase (*CRAT*) expression, suggesting that secretome addition does not affect lipid metabolism. It is known that amino acid metabolism supports oocyte growth and cytoplasmic maturation as mitochondria metabolize ketone bodies originating from deamination of certain amino acids such as leucine [10]. Interestingly, amino acid uptake by oocytes from old mares is limited compared to the ones retrieved from young mares, which is correlated with a loss in the reproductive performance with ageing, demonstrating its importance in the horse [34]. Moreover, a former study in bovine oocytes established that the determination of amino acid turnover can be used to obtain an index that predicts oocyte developmental competence in vitro [35]. In our study we observed that secretome addition induces an increase in threonine uptake in COCs obtained in vivo (OPU), but not in COCs obtained *post-mortem* (SLA). The secretome´s ability to modulate metabolism of target cells has also been previously demonstrated in breast cancer cells in which secretome from *Escherichia coli* significantly upregulated amino acid metabolism after 24 h post-treatment [36]. Interestingly, secretome from FF seems to enhance the metabolism of selected amino acids, acetic acid and glucose, differently in SLA and OPU retrieved COCs. The reason underlying why the effects of secretome differ depending on the oocyte´s source still needs to be determined, but our results may suggest that the ideal composition of IVM medium could vary depending on the COCs’ source (in vivo or *post-mortem*) and/or IVM onset (immediately vs. holding).

## Conclusions

This study shows the effects of secretome addition on equine COCs metabolism when added during IVM. While no effects on the meiotic competence of equine oocytes were observed, secretome addition increased the metabolism of glucose, as well as acetic acid and amino acid turnover in COCs obtained in vivo but not when obtained *post-mortem*. Furthermore, mRNA expression of *LDHA* was affected by secretome addition only in COCs obtained in vivo. More research is needed to fully understand why secretome addition during IVM influences differently the metabolic ability and relative mRNA expression of *LDHA* depending on the COCs’ source (in vivo or *post-mortem*).

## Materials and methods

### Chemicals and reagents

Unless otherwise specified, all reagents were purchased from Sigma Aldrich Quimica (Barcelona, Spain).

### Oocyte harvesting

Experiments were divided into two types of sessions depending upon oocyte retrieval source: in vivo by ovum pick up (OPU) or *post-mortem* obtained from a commercial slaughterhouse (SLA).

#### OPU sessions

A herd of nine mares housed at our institution were maintained according to European regulations. All experimental procedures were reviewed and approved by the Institutional Animal Care and Use Committee at the University of Extremadura and “Junta de Extremadura” (Ref. MAM/JSR). Mares underwent OPU by transvaginal aspiration once every two weeks. Sessions were performed when there was a minimum of ten follicles ≥ 5 mm diameter, which were aspirated as described previously [37]. Briefly, the mare was held in stocks, and a nonsteroidal anti-inflammatory drug (Flunixin Meglumine, Finadyne®, MSD Animal Health, Sint-Lambrechts-Woluwe, Belgium, 1.1 mg/kg, intravenous) and a broad-spectrum antibiotic were administered prior OPU. The sedation protocol comprised an initial bolus of xylazine hydrochloride (Xilagesic® 200 mg/ml; 0,8 mg/kg iv) and morphine (Morfina®, B Braun medical Rubı, Barcelona, Spain; 50 µg/kg iv). Sedation was maintained by a constant intravenous rate infusion of xylazine hydrochloride (0.65 mg/kg/h), morphine (30 µg/kg/h) and ketamine (Ketamidor® 100 mg/ml, Richer Pharma, Wels, Austria; 0.4 mg/kg/h) and an antispasmodic medication (N-butylscopolammonium bromide, Buscopan®, Boehringer Ingelheim, Brussel, Belgium, 0.3 mg/kg iv) was also provided. An operator positioned a 5-7.5 mHz sector ultrasound probe (LogiQ Scan, General Electrics, Madrid, Spain), fitted within a transvaginal probe handle with a needle guide (Boland Vet Sales, Keller, TX, USA), into the mare’s vagina. The ovary was grasped via transrectal palpation, and the probe was manipulated to visualize follicles in the ovary. A 12-gauge double-lumen needle (Minitube SL, Tarragona, Spain) was used to puncture the follicles and aspirate the follicular content using a vacuum pump (Cook, Limerick Ireland). When possible, each follicle was flushed six to eight times with EquiFlush embryo recovery medium with polyvinyl alcohol (PVA) and antibiotics (Ref. 19,982/6202, Minitube, Germany) containing 4 IU/ml heparin from porcine intestinal mucosa. The aspirated fluid was poured through a 70 μm nylon filter (Ref. 352,350, FALCON, Durham, NC, USA), and the contents rinsed with fresh flushing medium. COCs were located using a dissection microscope at 10× and washed twice before IVM.

#### SLA sessions

Ovaries were collected at a commercial slaughterhouse located 4 h away from our laboratory (Incarsa, Burgos, Spain). Transport was performed at 15 °C within 6 h of slaughter. Immature COCs were obtained using a vacuum pump (Cook, Limerick Ireland), in EquiFlush embryo recovery medium with PVA and antibiotics (Ref. 19,982/6202, Minitube, Germany) containing 4 IU/ml of heparin from intestinal porcine mucosa. COCs were washed twice and maintained in holding medium (40% M199 with Hanks salts, 40% M199 with Earle’s salts, 20% fetal bovine serum (FBS) and 25 µg/ml gentamicin) overnight at 20–24 ºC in the dark before IVM.

### Secretome isolation

The oestrous cycle of two healthy fertile mares was monitored by transrectal ultrasonography. When a follicle of at least 35 mm in diameter was detected in the absence of luteal tissue and with marked uterine oedema and low cervical tone, mares received 3000 IU of hCG intravenous. After 32 h, FF was retrieved by flank aspiration [38]. To remove red blood cells and debris, FF was centrifuged at 4 °C, 700 × g for 10 min. Clean FF was passed to a clean 50 ml conical tube and centrifuged again at 4000 × g, 20 min at 4 °C. Then, three millilitres of FF from both mares were pooled, diluted 1:1 in phosphate-buffered saline (PBS), filtered with a 0.2 μm syringe filter and centrifuged (4000 × g, 1 h at 4 °C) using a 10 K Amicon® Ultra-15 Centrifugal Filter Unit. Protein concentration of the retrieved secretome was measured using a DC Protein Assay (Bio-Rad Hercules, CA, USA) following the instructions provided by the supplier; secretome was then aliquoted and stored at -80 °C.

### IVM

For IVM, COCs from each session were split into three equal groups and matured in basal medium only (TCM-199, 10% FBS and 5 mIU/ml FSH, Control or CTR group), or in basal medium supplemented with secretome at 20 µg/ml (S20 group) or at 40 µg/ml (S40 group). These dosages were chosen based on our previous work [12]. All media were equilibrated for at least 2 h prior IVM, and COCs were then cultured in droplets of different maturation media at a ratio of 20 µl of medium per oocyte for OPU-retrieved oocytes and in 500 µl droplets for the SLA group, under mineral oil (NidOil ™, Nidacom, International, Mölndal, Sweden) at 38.2 °C in a humidified atmosphere of 5% CO_2_ in air, for 26–28 h.

### Evaluation of oocyte degeneration and maturation status

Equine oocytes were evaluated after IVM. First, cumulus cells of COCs were removed in PBS supplemented with 0.01% PVA (w/v) (PBS + PVA) and 0.4% hyaluronidase (w/v) by repeated pipetting using decreasing diameter stripping pipettes (Cook, Barcelona, Spain) until no granulosa cells were visualized on the zona pellucida. Denuded oocytes were then fixed in 4% formaldehyde in PBS + PVA for 12 h at 4 °C. Then, oocytes were thoroughly washed in PBS + PVA and stained with 2.5 µg/ml Hoechst 33342 at 37 °C for 10 min in the dark. Oocytes were then mounted on slides using glycerol and a coverslip, sealed with nail polish, and allowed to air dry. Oocytes were observed under a Nikon Eclipse 50i fluorescence microscope equipped with a mercury lamp and a 60X objective. Then, oocytes were classified based on DNA integrity and conformation as germinal vesicle (GV), metaphase I (MI) or MII, following previously validated criteria [29]. Oocytes were considered as degenerated (DEG) when no DNA was present or if unidentifiable chromatin configurations were observed.

### Metabolomic analysis of IVM media

The influence of secretome supplementation on COCs’ metabolism was studied by comparing the metabolic composition of IVM media before (Pre-IVM) and after (Post-IVM) maturation using nuclear magnetic resonance (NMR). At least 60 µl of pre- and post-maturation supernatants were recovered from each sample (Control, S20 and S40; SLA sessions *n* = 3 and OPU sessions *n* = 6) and kept at -80 °C until analysis. After thawing, the supernatants were subjected to proton NMR (^1^H-NMR) as described in our previous studies [4, 29].

#### Sample preparation

On the day of analysis, samples were thawed for 30 min slowly on ice. A total of 10 µl of sample was diluted in 189 µl of 0.2 M potassium phosphate buffer in deuterium oxide (D_2_O) with a pH of 7.4 ± 0.5 and 1.11 µl of TSP-d6 (3-(Trimethylsilyl)propanoic acid), to reach a final volume of 200.11 µl. Samples were vortexed for 10 s and added into a 3 mm NMR tube.

#### NMR measurements

Samples were measured at 300 K on a 600 MHz IVDr (Bruker BioSpin, Germany), with a thermostatised automatic sample changer (SampleJet) and a double resonance broadband probehead (BBI) with a z gradient coil and BOSS-III shim system.

Before sample acquisition, the spectrometer was calibrated with two different samples: methanol and sucrose to check the temperature (300 K) and optimal shimming respectively. Three main spectra were acquired for all the samples. Standard one-dimension 1 H NOESY spectrum (noesygppr1d) with water presaturation was acquired with 4 dummy (DS) and 32 accumulated (NS) scans. A 1D 1 H Carr-Purcell-Meiboom-Gill (CPMG) experiment (cpmgpr1d) was acquired with 4 DS and 256 NS, and a two-dimensional J-resolved experiment (jresgpprqf) was acquired to help on the signal multiplicity identification. Spectra were acquired and processed using the TopSpin 3.6.2 software (Bruker Biospin GmbH). Free induction decays were multiplied by an exponential function equivalent to 0.3 Hz line broadening before applying Fourier transform. All transformed spectra were corrected for phase and baseline distortions and referenced to the TSP singlet at 0 ppm.

The concentrations of the identified metabolites are expressed in micromolar units. The metabolites’ concentration was calculated relatively to integrate the signal of each metabolite and the number of protons associated with that signal. Metabolite concentration was then extrapolated using TSP singlet at 0 ppm. The metabolites were determined following previously validated methods (Table 3) [4, 39].

**Table 3:** Chemical shift assignment, multiplicity, and number of contributing protons for the identified metabolites

Metabolites	Peak for integration (ppm)	Multiplicity	Number of protons
Lactic acid	4.1	q	1
Glucose	5.23	d	1
Tyrosine	6.9	m	2
Glycerol	3.64	m	4
Succinic acid	2.389	s	4
Pyruvic acid	2.363	s	3
Glutamic acid	2.335	m	2
Acetic acid	1.906	s	4
Alanine	1.47	d	3
Threonine	1.316	d	3
3-hydroxyisovaleric acid	1.237	d	3
Valine	1.03	d	3
Isoleucine	1.004	d	3
Leucine	0.96	t	6

### RNA extraction, reverse transcription, and quantification of mRNA transcript abundance

Gene expression was analysed in matured COCs from the different IVM treatments retrieved on eight different sessions (SLA COCs: *n* = 25 for CTR, *n* = 25 for S20, *n* = 25 for S40, and OPU COCs: *n* = 20 for CTR, *n* = 26 for S20, *n* = 23 for S40). After IVM, COCs were thoroughly washed in PBS + PVA, placed in 10 µl of sterile PBS, plunged into liquid nitrogen and stored at -80 °C until RNA extraction [40]. Before RNA extraction, samples were thawed and Poly(A) RNA was extracted using a Dynabeads mRNA DIRECTMicro Kit (Ambion; Thermo Fisher Scientific), as described previously [41]. Briefly, samples were incubated in lysis buffer for 10 min with Dynabeads, poly(A) RNA attached to the Dynabeads was extracted magnetically and washed twice with Washing Buffer A and Washing Buffer B. RNA was obtained after elution with Tris-HCl. The reverse transcription reaction was then performed according to the manufacturer’s instructions (Epicentre Technologies); to prime the reverse transcription reaction and to produce cDNA poly(T) primers, random primers and MMLV High Performance Reverse Transcriptase enzyme were mixed in a total volume of 40 µl. Tubes containing the mixture were heated to 70 °C for 5 min to denature the secondary RNA structure and, after the addition of 50 units of reverse transcriptase, the reverse transcription reaction was completed. Retrotranscription was performed by incubating samples at 25 °C for 10 min to favour the annealing of random primers, followed by incubation at 37 °C for 60 min to allow the reverse transcription of RNA and a final incubation at 85 °C for 5 min to denature the enzyme. After cDNA synthesis, samples were made up to 55 µl using 10 mM Tris-HCl (pH 7.5).

Three cDNA samples were used per experimental group and all qPCR reactions were performed in duplicate using the Rotorgene 6000 Real Time Cycler (Corbett Research). The relative levels of each transcript in each sample were normalised against that of the housekeeping gene histone H2A family, member Z (*H2AFZ*). The PCR was performed by adding a 2 µl aliquot of each cDNA sample to the PCR mix (GoTaq qPCRMaster Mix; Promega) containing specific primers selected to amplify the selected genes, namely Lactate dehydrogenase A (*LDHA*), Solute carrier family 2 member (*SLC2A*), Glyceraldehyde-3-phosphate dehydrogenase (*GAPDH*), Fatty acid binding protein 3 (*FABP3*) and Carnitine acetyltransferase (*CRAT*). Primers were designed using Primer-BLAST software (https://www.ncbi.nlm.nih.gov/tools/primer-blast/) to span exon–exon boundaries when possible (Table 4). Cycling conditions were as follows: 94 °C for 3 min, followed by 35 cycles of 94 °C for 15 s, 56 °C for 30 s and 72 °C for 10 s, with a final 10 s for fluorescence acquisition. Each pair of primers was tested to achieve efficiencies close to 1; to quantify expression levels of the target genes, the comparative cycle threshold (CT) method was used [42]. Fluorescence was acquired in each cycle at a temperature higher than the melting temperature of primer dimers to avoid primer artefacts (specific temperatures for each product varied from 80 °C to 86 °C). For each sample, the CT or the cycle during the logarithmic linear phase of the reaction at which fluorescence increased above background was determined. The ∆CT value was determined by subtracting the quantification cycle (C_q_) value of the endogenous control (*H2AFZ*) for each sample from the C_q_ for each gene in the sample. Determination of ∆∆CT involved using the highest sample ∆C_q_ value as a constant to subtract from all other ∆Cq sample values. Fold changes in the relative expression of target genes was determined as 2 – ∆∆C_q_.

**Table 4:** Details of primers used for qRT-PCR analysis

Gene	Gene name	Primer sequence (5’- 3’)	Fragment size (bp)	GenBank accession number
*H2AFZ*	H2A histone family, member Z	AGGACGACTAGCCATGGACGTGTG CCACCACCAGCAATTGTAGCCTTG	209	NM_174809
*SLC2A1*	Solute carrier family 2 (facilitated glucose transporter), member 1	CACTGGAGTCATCAACGCCC CCACGATCAGCATCTCAAAG	287	XM_005607003.3
*LDHA*	Lactate dehydrogenase A	CCGTGTTATCGGAAGTGGTTGC AGAATCTCCATGCTCCCCAAGG	121	NM_001144880
*GAPDH*	Glyceraldehyde-3-phosphate dehydrogenase	GAGATCAAGAAGGTGGTGAAGC CATCGAAGGTGGAAGAGTGG	121	NM_001163856.1
*CRAT*	Equus caballus carnitine O-acetyltransferase	GCCATTGCCATGCACTTCAACCTC AGGTGGCTTGGCTGTGGCATTG	295	XM_023629103.1
*FABP3*	Equus caballus fatty acid binding protein 3	CCTACTGGCTCCTTCACTGAC AGAACTTCCCCAACCAAGCTG	119	NM_001163885.2

### Statistical analysis

All statistical tests were performed using Sigma Plot 12.0 for Windows (Systat Software. Chicago. IL. USA). To assess normality, a Saphiro-Wilk test was used and to test the equality of variances, a Levene´s test was performed. Data for oocyte’s chromatin configuration showed normal distribution and homogeneous variances, thus, they were compared using a one-way analysis of variance (ANOVA) followed by Dunn´s post hoc test. Data for relative mRNA abundance and NMR that followed a normal distribution and had homogeneous variances were compared using an ANOVA followed by Fisher LSD method post hoc test. If data followed a normal distribution but failed the equal variance test or presented a non-gaussian distribution, they were compared using the non-parametric Kruskal Wallis test. Pairs of values for NMR assays pre-IVM and post-IVM within the same group (Control, S20 or S40) were compared using a Student’s t-test. Values were considered significantly different when the p-value was < 0.05. Unless otherwise indicated, data are presented as the mean ± standard error of the mean (SEM).

### Electronic supplementary material

Below is the link to the electronic supplementary material.

Supplementary Material 1

Supplementary Material 2

## Data Availability

No datasets were generated or analysed during the current study.

## Abbreviations

ARTs: Assisted reproductive technologies
COC: Cumulus-oocyte complex
DEG: Degenerated
EVs: Extracellular vesicles
FF: Follicular fluid
GV: Germinal vesicle
ICSI: Intracytoplasmic sperm injection
IVEP: In vitro embryo production
IVF: In vitro fertilization
IVM: In vitro maturation
MI: Metaphase I
MII: Metaphase II
NMR: Nuclear magnetic resonance
OPU: Ovum pick up
Pre-IVM: Prior in vitro maturation
Post-IVM: After in vitro maturation
S20: secretome from preovulatory follicular fluid at 20 µg/ml
S40: secretome from preovulatory follicular fluid at 40 µg/ml
SLA: Slaughterhouse
