# Supplementary figures and images for: Preovulatory follicular fluid secretome added to in vitro maturation medium influences the metabolism of equine cumulus-oocyte complexes
Source: BMC Vet Res. 2024 Jun 25;20:272. doi: 10.1186/s12917-024-04129-1 (PMC11197253; doi:10.1186/s12917-024-04129-1)

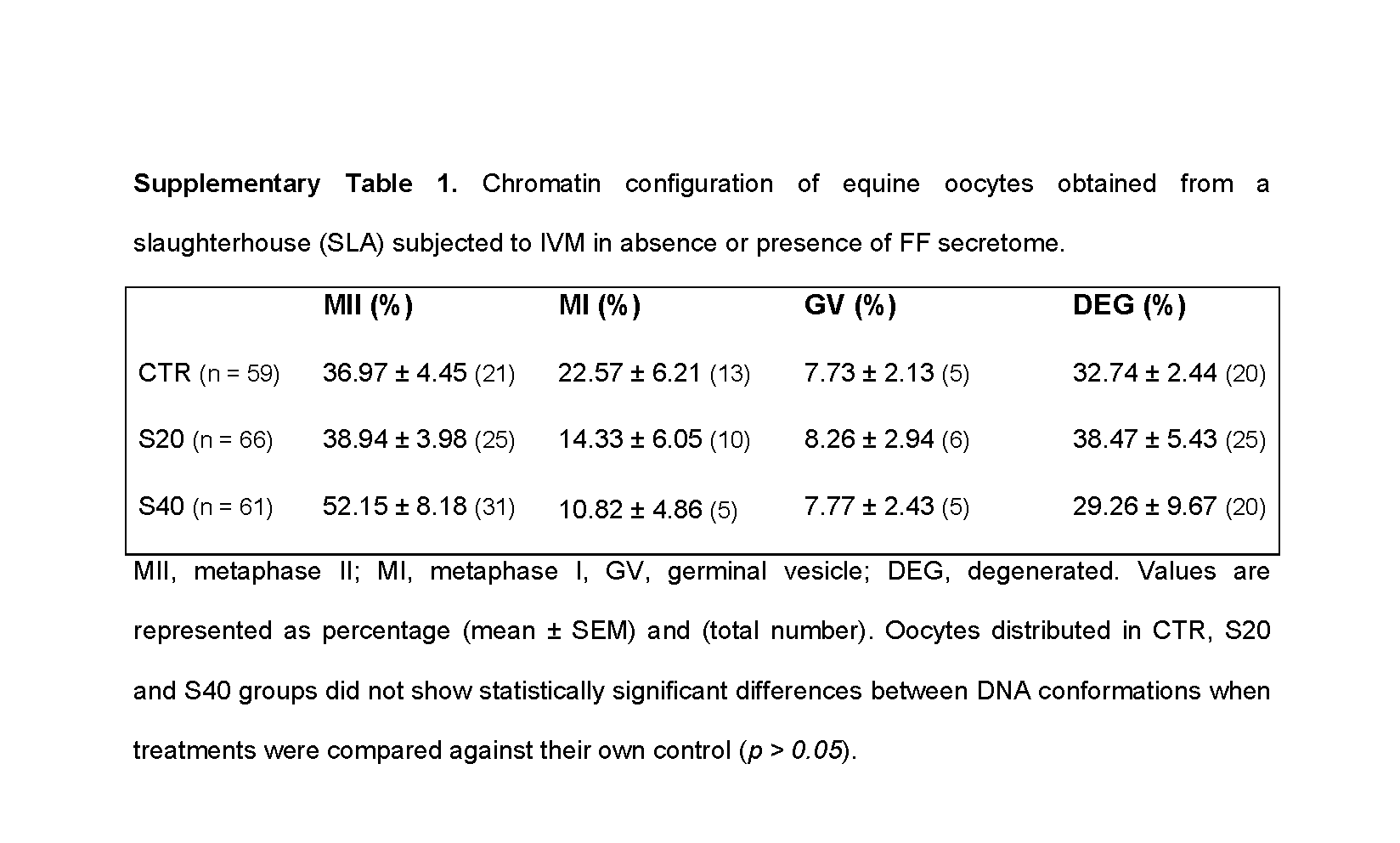

Supplement: Supplementary file 1 — Supplementary Material 1 [file 12917_2024_4129_MOESM1_ESM.tiff]

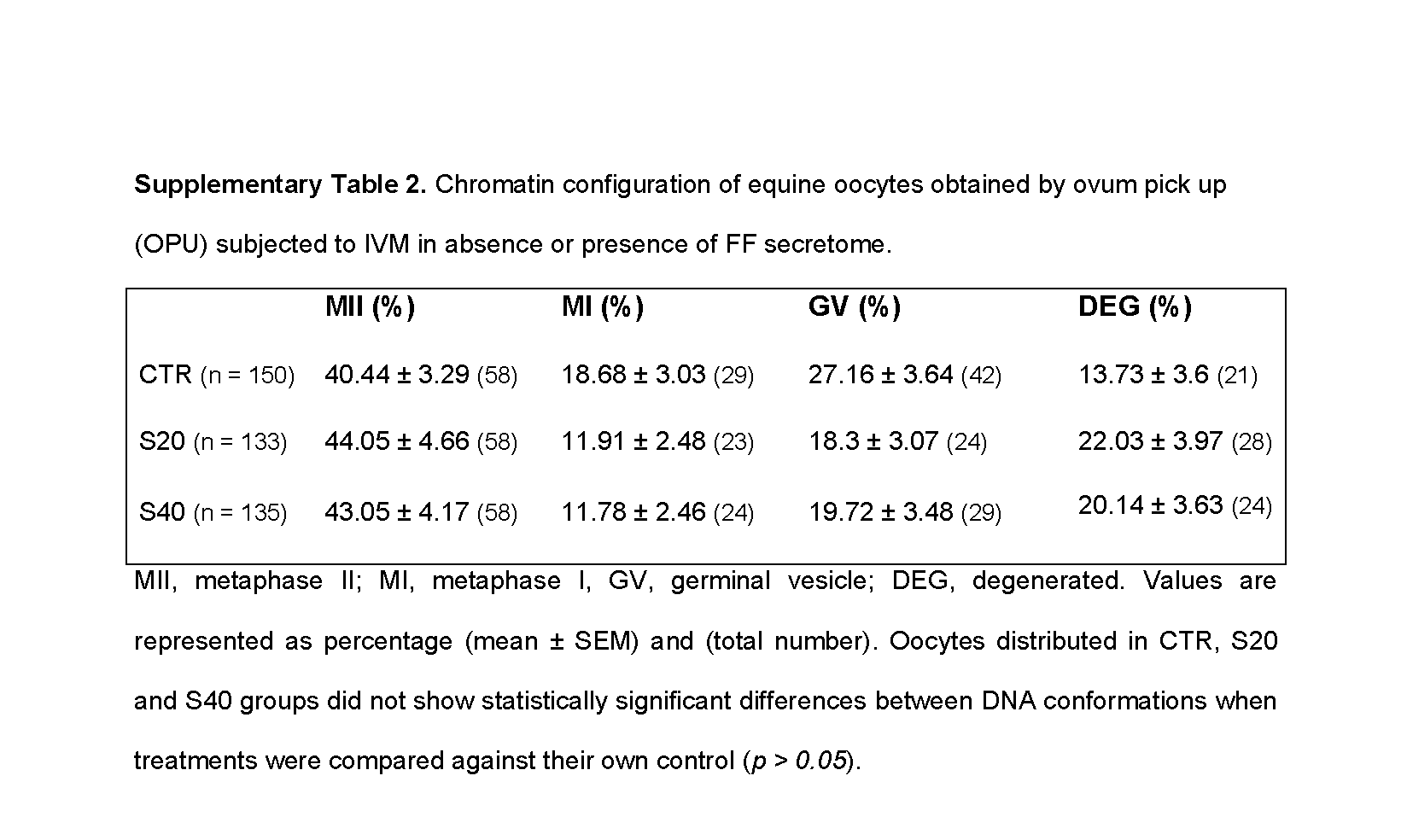

Supplement: Supplementary file 2 — Supplementary Material 2 [file 12917_2024_4129_MOESM2_ESM.tif]
